# The Human African Trypanosomiasis Specimen Biobank: A Necessary Tool to Support Research of New Diagnostics

**DOI:** 10.1371/journal.pntd.0001571

**Published:** 2012-06-26

**Authors:** Jose R. Franco, Pere P. Simarro, Abdoulaye Diarra, Jose A. Ruiz-Postigo, Jean G. Jannin

**Affiliations:** 1 World Health Organization, Control of Neglected Tropical Diseases, Innovative and Intensified Disease Management, Geneva, Switzerland; 2 World Health Organization, Regional Office for Africa, Brazzaville, Congo; 3 World Health Organization, Regional Office for the Eastern Mediterranean, Cairo, Egypt; Institute of Tropical Medicine, Belgium

## Background

Human African trypanosomiasis (HAT), or sleeping sickness, is a vector-borne disease caused by trypanosomes (*Trypanosoma brucei gambiense* and *T.b. rhodesiense*) mainly affecting impoverished rural areas in sub-Saharan Africa, where the health systems are weak.

Over the last decade, the number of HAT cases has shown a decreasing trend as a result of coordinated control efforts [1]. This makes it possible to envisage the elimination of the disease, but a new approach to uphold current results is needed. Sustainability of the control efforts will require integration of control and surveillance activities within a reinforced health system [2]. However, the complexity of the existing diagnostic tools is not compatible with prevailing conditions at basic health facilities in rural areas where the disease is endemic, which hinders the participation of the health system in the control and surveillance of the disease [3]. There is an urgent need for diagnostic tests that are reliable, cheap, and easy to perform at basic health services.

In 2006, the Department of Control of Neglected Tropical Diseases (NTD) of the World Health Organization (WHO) established a collaboration with the Foundation for Innovative New Diagnostics (FIND, http://www.finddiagnostics.org/) to develop new diagnostic tools for the control of HAT that meet the requirements of a sustainable elimination approach. In the framework of this agreement, WHO established a HAT specimen biobank as a collection of biological specimens related to HAT, coupled with clinical and epidemiological information of the person who donated the specimens. The specimen biobank is the property of WHO and its main objective is to provide clinical reference material to research institutions to facilitate the development and evaluation of new tests for the diagnosis of HAT.

To set up a specimen bank for HAT first requires the collection of specimens while strictly following good clinical practice principles. The specimens have to be collected in the areas where the disease is endemic, usually remote areas with limited health resources and impoverished affected populations. The specimens collected have to be well identified and kept in strict cold chain from the time of collection to the final storage. To fulfill these conditions is challenging, but we have proved that it is not insurmountable.

## What Are the Characteristics and Requirements of the HAT Biobank?

The WHO HAT biobank includes specimens from three groups of participants:

- *Cases*, defined as individuals where presence of trypanosomes was confirmed.
- *Controls*, defined as individuals living in endemic areas with negative serology (Card Agglutination Test for Trypanosomiasis, CATT) and parasitology for HAT, and without evidence of previous HAT infection.
- *Suspects*, defined as individuals with positive serology for HAT but negative parasitology and no evidence of previous HAT infection. According to national protocols, these individuals usually do not receive treatment but are followed-up until confirmation or rejection of the serological suspicion. They were also asked to participate during their follow-up (at least one visit), with specimens taken during each follow-up visit.

All participants were ≥12 years old, and were enrolled only after giving written informed consent. Informed consent forms were prepared in different local languages (Kiswahili, Lingala, Chiluba, Ngambaye, Kakwa, Kumam, and Lugbara). For patients unable to give consent due to HAT-related mental impairment and for participants under 18 years old, provision of informed consent was done by the legal guardian (with an informed assent signed by the minor).

Specimens collected from each donor include blood, serum, plasma, saliva, and urine. In Cases, cerebrospinal fluid (CSF) was also taken. These specimens were obtained during routine examination of the participants. Clinical, epidemiological, and laboratory data were recorded and linked to biological specimens.

Specimens are strictly kept below −80°C from collection to delivery to the final users. The cold chain was based on immediate storage in liquid nitrogen after collection, and intermediate storage in the national centers in liquid nitrogen or in ultra-low deep freezers while waiting for shipment on dry ice to the Central Repository by express courier.

The specimen collection and banking was approved by the WHO Ethical Review Committee and the different national ethical committees in each country where specimens were collected. The national Ministries of Health also gave their approval.

## Where Were the Specimens Collected?

**Figure 1:**
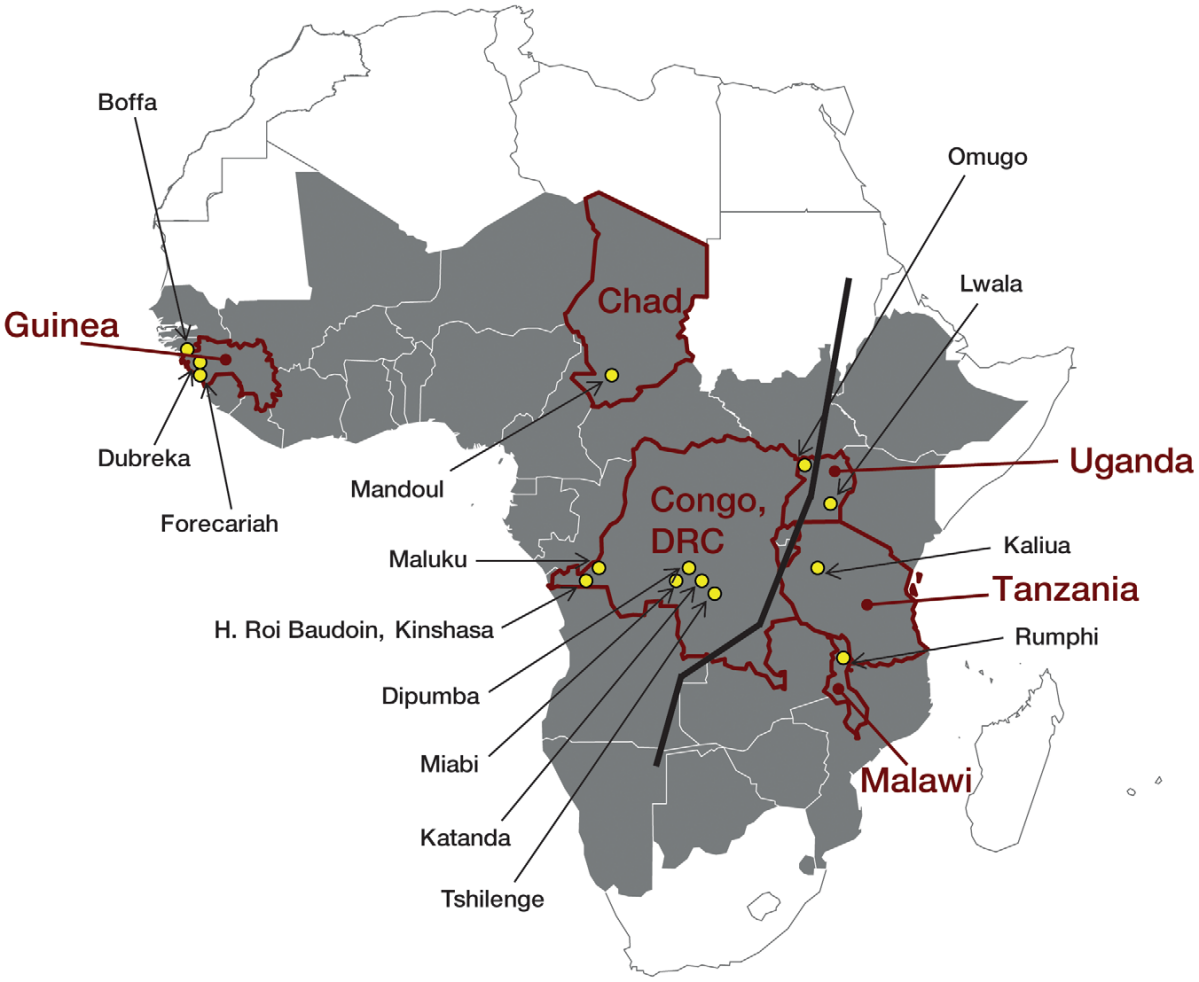
Localities of collection sites of specimens for the HAT specimen biobank (yellow dots). Dark grey indicates HAT-endemic countries. The bold black line shows the theoretical separation of *T.b. gambiense* and *T.b. rhodesiense* areas.

Collection sites were selected according to accessibility, security, communication, number of cases and subspecies of HAT reported (*T.b. rhodesiense* and *T.b. gambiense*), and available facilities, equipment, and human resources as well as existing support from recognized national institutions. The sites selected for the collection and the national institutions for intermediate storage were as follows (Figure 1):

- Democratic Republic of the Congo (DRC) in partnership with the Sleeping Sickness National Control Program (SSNCP) and in collaboration with the National Institute for Biomedical Research (INRB): Two sites in Kinshasa province (“Hôpital Roi Baudouin” in Kinshasa and “Centre de Diagnostic et de Traitement de la Maladie du Sommeil” [CDT] in Maluku) and four sites in Kasai Orientale province (the CDT of Dipumba, the CDT of Katanda, the “Unite Mobile” [UM] of Miabi, and the UM of Tshilenge).
- United Republic of Tanzania, in partnership with the National Institute for Medical Research (NIMR): Kaliua Health Centre in Urambo District.
- Guinea, in partnership with the SSNCP and in collaboration with the Institut de Recherche pour le Développement/Centre International de Recherche - Développement sur l'Elevage en zone subhumide (IRD/CIRDES): UM working in the HAT foci of Forecariah, Dubreka, and Boffa.
- Malawi, in partnership with the SSNCP and the Centre for Tick and Tick-borne Diseases (CTTBD): Rumphi Hospital in Rumphi district.
- Chad, in partnership with the SSNCP and the Organisation pour le Control des Endémies en Afrique Central (OCEAC): UM working in the Mandoul focus (Préfecture de Bodo).
- Uganda, in partnership with the SSNCP and the University of Makerere: Omugo Health Centre IV in Maracha-Terego District, and Lwala Hospital in Kaberamaido District.

Collection of specimens started in 2008 and eventually 949 Cases (113% of total planned), 759 Controls (91% of total planned), and 90 Suspects (90% of total planned) were enrolled (1,798 participants) (Table 1).

**Table 1:** Number of participants enrolled (by country).

	Cases	Controls	Suspects	TOTAL
**Planned**	840	840	100	1,780
**Collected (performance)**	949 (113%)	759 (90%)	90 (90%)	1,798 (101%)
Guinea	90	30	42	149
DRC	598	597	48	1,261
Tanzania	18	36	0	55
Malawi	73	70	0	143
Chad	73	26	0	99
Uganda (*T.b.g.*)	50	0	0	47
Uganda (*T.b.r*.)	47	0	0	50

*T.b.g.*, *T.b. gambiense*; *T.b.r.*, *T.b. rhodesiense*.

## How Were the Specimens Shipped and Stored?

Following an open tender, the “Clinical Investigation and Biomedical Research Support Unit (ICAReB)” of the Institut Pasteur in Paris was selected as Central Repository. The Central Repository is in charge of management of specimens and associated data, including reception, control, processing, storage, and subsequent distribution to end-users, following all applicable quality standards and regulations. ICAReB obtained all due approvals from the French Research Ministry and Ethical Committee Ile-de-France I.

Shipment from intermediate national storage to the Central Repository was organized by express courier on dry ice. A total of 18 shipments were made: seven from DRC, two from Tanzania, two from Chad, two from Guinea, three from Malawi, and two from Uganda.

Specimens from 1,804 participants originally enrolled arrived at the Central Repository and were processed and stored (Table 2). Specimens from 157 participants (9%) are currently in quarantine due to lack of key information or inconsistencies in the data forms.

**Table 2:** Summary of specimens stored in ICAReB, Institut Pasteur, Paris.

Received	Controls (C)	Cases (P)			Suspects (S)	
		Stage 1 (P1)	Stage 2 (P2)	Stage X (PX)	First (S1)	Follow-Up (Sx)
*T.b. gambiense*	653	182	626	3	90	29
*T.b. rhodesiense*	106	16	95	27	—	—
TOTAL	759	198	721	30	90	29
Total by category	759		949		90	29
TOTAL INDIVIDUALS			1,798			29

P1, case stage 1; P2, case stage2; Px, case stage not determined; C, controls; S1, suspects' initial visit; Sx, suspects' follow-up visits.

Quality control of the specimens stored in the Central Repository has been performed by the WHO collaborating centre for HAT diagnosis based at the Institute of Tropical Medicine in Antwerp. A CATT dilution was performed, followed by the immune trypanolysis test [4], when discordance in CATT results with results from the field was observed. Specimens from eight participants were discarded during this quality control process.

## How Are the Specimens Distributed?

Distribution of the specimens is limited to qualified investigators involved in the development and evaluation of new diagnostics for HAT that would be appropriate for use in low-income countries and will benefit affected populations.

A material request form is available at the WHO website (http://www.who.int/trypanosomiasis_african/research/en/) and should be completed to request specimens. With this form, the institution provides specific information on the intended use of the specimens, and accepts the general conditions of use. An Exit Committee examines the request received on pertinence, relevance, and coherence with the objectives of the biobank. If the request is accepted, exit orders are given by WHO to the Central Repository, which organizes the shipments.

To date, the Exit Committee has received 15 requests for specimens from research institutions in the United States, Switzerland, Belgium, United Kingdom, Spain, Germany, Korea, Kenya, and France. A total of 2,890 specimens (1,163 serum, 1,336 plasma, 10 buffy-coat, and 381 CSF) have been supplied to research institutions based on the recommendations of the Exit Committee decisions. The average time from receiving the request to Exit Committee decision was 15 days (5–49 days). The average delivery time after Exit Committee decision was 48 days (28–120 days).

Request of specimens from the WHO HAT biobank can be addressed to WHO/NTD (francoj@who.int, simarrop@who.int) or by consulting: http://www.who.int/trypanosomiasis_african/research/en/.

**Learning Points**

- To set up a specimen biobank is an expensive and complex task [5]–[7]. These problems are increased when the subject of the biobank is a neglected disease such as HAT, occurring in remote impoverished areas. Despite logistical, technical and financial difficulties, the HAT biobank has now been set up and is functional. A large collection of specimens is available for research on new diagnostic tools. Collection of the specimens has respected ethical principles and adhered to good clinical and laboratory practice.
- Collaboration of SSNCP and research institutions has been essential to set up the biobank.
- As collateral benefits, the WHO specimen biobank has also helped to improve the skills of involved staff, to strengthen the diagnostic capacity in screening sites, to reinforce current control and surveillance activities (screening, logistics, and mobile teams), to upgrade equipment of institutions involved in HAT screening, and to train on ethical aspects of the research.
